# Supplementary material for: Patterns of Intron Gain and Loss in Fungi
Source: PLoS Biol. 2004 Nov 30;2(12):e422. doi: 10.1371/journal.pbio.0020422 (PMC532390; doi:10.1371/journal.pbio.0020422)
Supplement: Table S1 — Also available at http://genes.mit.edu/NielsenEtAl/. (4.3 MB ZIP). [file pbio.0020422.st001.zip › NielsenEtAl/html/376.html]

AN2140.1.NCU01146.1.MG07209.1.FG07412.1


```
 CLUSTAL W (1.82) Multiple Sequence Alignments - Introns Inserted


Sequence 1: NCU01146.1	272 aa
Sequence 2: FG07412.1	265 aa
Sequence 3: MG07209.1	267 aa
Sequence 4: AN2140.1	259 aa
Alignment Length: 274 aa
Number Identitical Residues: 71 aa
Alignment Score (without introns) 4563


MG07209.1 	---MVSLKLTGLAMLAFQLMGAFA~Q~AE0GTAEPQTPAEPQLKLDVATTFPDADIFGVK
NCU01146.1	MVNFRWCSLALLALRAVSVFAQEE0T~FG~DDSTAAPVSQPELKADIETTFPDADIFGVK
FG07412.1 	----MFFKASLLALAASHIFGAVA~Q0DA~PDANAAPKAP--LKAEIKTTFPETDILGVK
AN2140.1  	MARFAFLSLALFSVQAL-IGGALA~A0DT~ADKAEETFEAPTLAVTAQAAFPASEIFGVK
          	       . : ::: *  : .               .   . *     ::** ::*:***

MG07209.1 	LVNGRPTKAVVDLTNNEADPVRLALVTGALTNPDGLAEGAPVSDAIIRNLTAVKYDVEIP
NCU01146.1	LVNGKVTKALIDITNQEDGPIQVAFVGGALKTTRPLPEDAPVYAQIVRNLTATRFQIEIP
FG07412.1 	LVNGRPTKAVIEITNQDANPIQVSFVSGSLNTLKDLPEDSPRYASIVRNLTAVQYNIEVP
AN2140.1  	LVNGHPTQALVTFTNNEKSAVTVNFIGGTLST---LGEES----KLVRNLTATRYGVEIP
          	****: *:*:: :**:: ..: : :: *:*..   * * :     ::*****.:: :*:*

MG07209.1 	AGEKRSVTYSFALDMQPRDVVVELLAIVINAKGQTFQVPAHADKASVVEPPTSFFDPQI2
NCU01146.1	AGEKQTLPYNFVLDMMPQDVVVELLAVVTNPAGEVFEIQLHNGTASIVEAPTSIFDPQI2
FG07412.1 	AGEKKELPFSFAQDMQPQEVRIELQALVSDSTGALHEVLVHNGSASIVEAPTSFLDPQI2
AN2140.1  	AGAQESLSYSFATEMHPQDLRLSLASVVSDTEGRFFTVYAYNGTVSVVEPETSIFDPQI2
          	** :. :.:.*. :* *::: :.* ::* :. *  . :  : ...*:**. **::**** 

MG07209.1 	IFLYLVLSAAFAGTLYFVYKTWIEALFPQAKRPRAQQPKKVVKVEAAEPLSGNESAGAAS
NCU01146.1	IFLYLFLTGVFGATLYFVYKTWIEALFPQAVKKTKA-PKVKK-VEVSEPLSGSESAGATS
FG07412.1 	IFLYLFLSAAFAGTLYFVYKTWIEALFPQAKRPRTN-KKAKKTVDVAEPLSGSEAVPVVD
AN2140.1  	IFLYFFLLACFGGVVYFFYTVWIAPYFPQKRKSAKQ-ETSRKNVASKKTEASVDSPAVSS
          	****:.* . *...:**.*..** . ***  :      .   .*   :. :. ::  . .

MG07209.1 	G--ADFDAKWIPDHHINRPVAKRVKSSASAKAKKGE--
NCU01146.1	GGEKNFDESWIPSHHLNRPVARRVKSGASAKAKKGGDQ
FG07412.1 	ASGKDYDESWIPDHHINRPVARRVKSSASAKAKRVE--
AN2140.1  	A--TTYNAEWIPAHHINRPEARKVKGTSRSKSRA----
          	.    :: .*** **:*** *::**. : :*::
```
